# A Study to Assess the Awareness and Attitudes Towards Artificial Intelligence (AI) Tools Among Medical Trainees and Practitioners

**DOI:** 10.7759/cureus.109790

**Published:** 2026-05-28

**Authors:** Kirti Chouhan, Khushbu Jangir, Medha Mathur

**Affiliations:** 1 Department of Community Medicine, Geetanjali Medical College and Hospital, Udaipur, IND

**Keywords:** artificial intelligence, ethics, health professionals, img, medical education

## Abstract

Background: Artificial intelligence (AI) is increasingly integrated into healthcare and medical education, offering support in clinical decision-making, learning, and information retrieval. However, its effective adoption depends on users’ awareness, perception, and readiness. The study aimed to evaluate awareness and utilization of AI tools, assess perceptions, and identify facilitators and barriers influencing their acceptance among healthcare professionals and medical students.

Methods: A cross-sectional study was conducted from September 2025 to January 2026 at a tertiary care center in Southern Rajasthan among 377 participants, including consultants, postgraduate trainees, and undergraduate students. A semi-structured, pre-validated, web-based questionnaire was used.

Results: Among all, 327 (86.7%) participants were aware of AI bots, while only 51 (13.5%) had received formal training. Despite this, 302 (82%) reported using AI tools for academic and clinical purposes, with 93 (24.7%) using them daily. Common uses included clinical decision-making, studying medical concepts, and preparing presentations. Participants’ primary concerns included accuracy (190, 50.4%), impact on critical thinking (235, 62.3%), and interpretation challenges (155, 41%). Major barriers identified were a lack of awareness, reliability issues, fear of academic misconduct, poor internet connectivity, and a lack of faculty endorsement. Facilitators included institutional guidelines, faculty training, workshops, mentorship, and curriculum integration.

Conclusion: AI tools are widely used among healthcare professionals and students despite limited formal training. While perceived as useful and time-saving, concerns regarding reliability, ethics, and overdependence persist. Strengthening training, developing clear guidelines, and integrating AI into medical curricula are essential to ensure safe and effective utilization.

## Introduction

Artificial intelligence (AI), a branch of machine learning capable of crafting new content in a variety of forms like text, images, audio, computer code, and video, is finding applications in many fields [1]. AI is becoming a useful part of healthcare as it’s gaining significant attention for its potential to support both clinical practice and medical education [2]. In the growing world of AI bots that can understand and respond to human language and help in clinical decision-making, learning, patient communication, and information retrieval, the dependence of medical health professionals has increased [3]. The integration of AI in healthcare is no longer a futuristic concept but a present reality. AI has the potential to improve diagnostic accuracy and reduce the workload of healthcare professionals. However, the success of these tools depends heavily on the readiness of the people using them and their perception of this digital shift [4,5].

As these AI bots become more common, it is important to understand how healthcare professionals are using them, what they think about their usefulness, accuracy, and role in daily work, as well as about overdependence, reliability, and confidentiality.

This research aims to study the awareness of and dependence on AI bots in clinical and academic settings. The objectives of the study are to evaluate the awareness of AI tools among healthcare professionals and medical students; to assess the perception of AI tools among healthcare professionals and medical students; and to identify the facilitators and barriers that influence healthcare professionals' acceptance and dependence on AI tools.

By assessing the gap between perception and actual uses, as well as the benefits, risks, and barriers of AI adoption, this research intends to provide information on AI utilization among medical practitioners and trainees at a tertiary care center in Southern Rajasthan.

## Materials and methods

This cross-sectional study was conducted at Geetanjali Medical College and Hospital, Udaipur, a tertiary care center of Southern Rajasthan, and the study participants were medical trainees, including undergraduate and postgraduate students and consultants serving in the healthcare facility. The study was conducted from September 2025 to January 2026.

The sample size was determined using the formula: $n=\frac{Z^2 \cdot p(1-p)}{d^2}$ where n is the sample size; Z is a standard normal variate at 5% type I error, its value is 1.96; p is the prevalence rate as per previous data, and d is the margin of error.

As per a previous study, the prevalence of AI awareness in medicine was found to be 26% as per previously published data, and the absolute error of precision was considered as 5% [3]. So, the estimated sample size was calculated to be 296, and considering the non-responders, an additional 10% sample was taken as 325.

A non-probability convenience sampling technique was used for data collection. Participants were recruited based on their accessibility and willingness to participate. The data were collected using a semi-structured, pre-validated, self-administered web-based questionnaire (Appendix 1). The questionnaire was developed and validated by experts from the institute and outside the institute. The questionnaire was circulated via digital platforms. Data was collected till the desired sample size was achieved. The questionnaire had details about the demography of participants, awareness and use of AI tools, perception towards AI tools, and barriers and facilitators of AI tools. The responses were in the five-point Likert scale format (ranging from strongly agree to strongly disagree), and some responses were open-ended to assess the perception of respondents regarding AI bot use.

The trainees and practitioners responded after giving consent to participate in the study. The ethical clearance of the study was obtained from the Institutional Ethics Committee numbered GU/HREC/EC/2025/2743, dated 29/09/2025. The participants who gave their consent to participate and were more than 18 years of age were included, while those who did not have electronic devices like smartphones/tablets or laptops and lacked access to internet connectivity were excluded.

After the data cleaning and coding, it was entered into MS Excel (Microsoft Corporation, Redmond, Washington, United States). Descriptive statistics, such as mean, median, standard deviation, and frequency distributions, were computed to summarize the key characteristics of the variables. Analysis was done using IBM SPSS Statistics for Windows, Version 26 (Released 2018; IBM Corp., Armonk, New York, United States).

Operational definitions

AI chatbot is a software that interacts and provides AI-based responses to the queries posed by the user. Facilitators are factors or conditions that promote, support, and enhance the effective and ethical use of AI tools among participants. Barriers refer to factors or challenges that hinder, limit, or discourage the adoption and appropriate use of AI tools among participants.

## Results

A total of 377 participants were included in the study. The response rate was 37%. Out of the total, 167 (44.3%) were males, while 210 (55.7%) were females. The mean age of the participants was 23.5 ± 5.9 years. The data were collected from 40 (10.6%) consultants, 134 (35.5%) postgraduate trainees, and 203 (53.8%) undergraduate students.

It was found that 327 (86.7%) participants were aware of AI bots, but only 51 (13.5%) were trained formally for using the AI in research and clinical practices, while 326 (86.5%) were using AI without any training.

**Figure 1:**
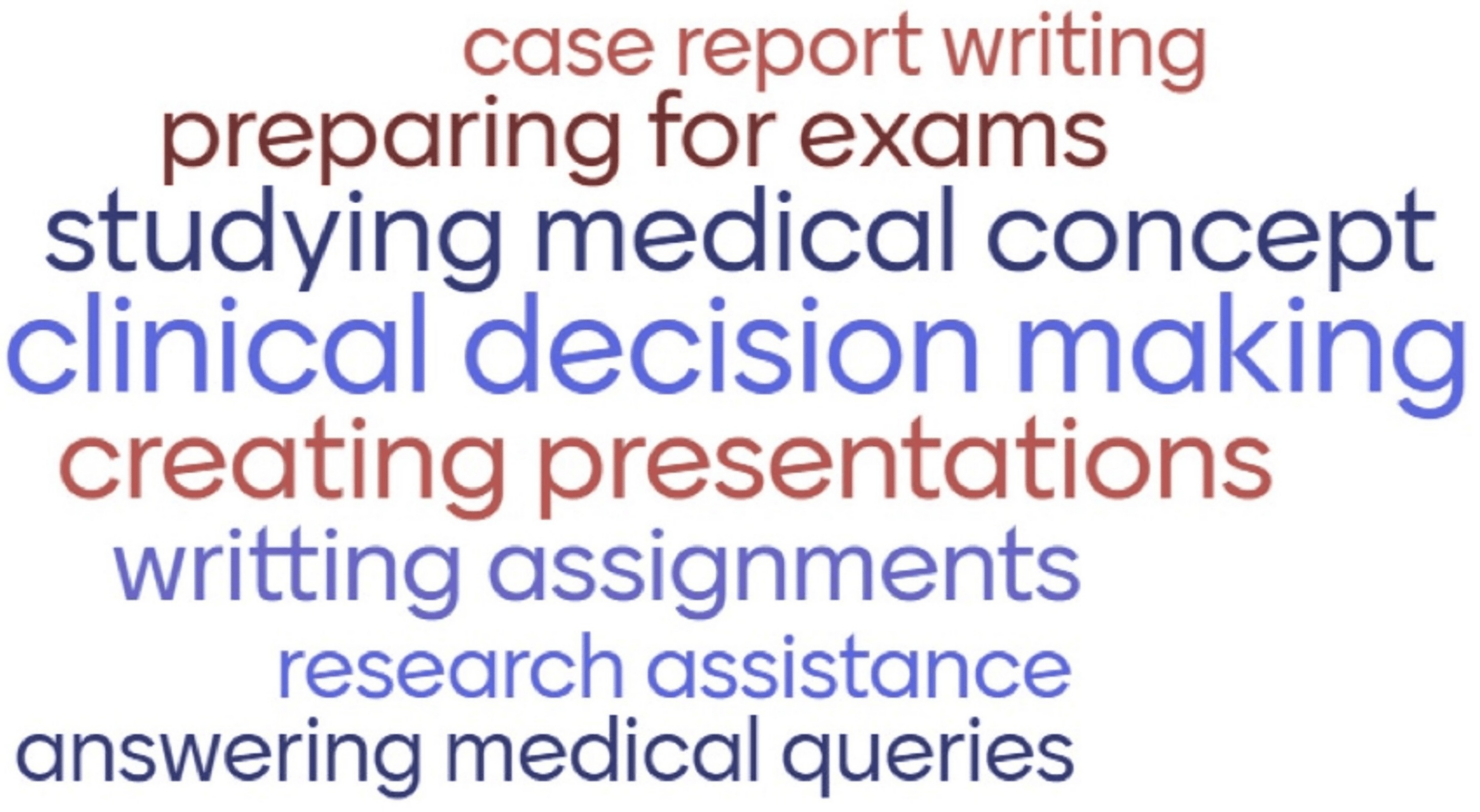
Word cloud depicting the uses of AI bots by participants

The majority of participants, 302 (82%), were found to have used AI bots for academic and clinical purposes. And it was found that one fourth (24.7%) of participants used the AI bots on a daily basis, 20.2% reported using them on a weekly basis, whereas 55.1% used them rarely. The various purposes for using AI bots are depicted in Figure 1 in word cloud format, where the size of the font of the response depicts the higher frequency of the result, which shows that the most common purpose of using AI bots was to make clinical decisions, followed by studying medical concepts and making presentations. Other responses included preparing for exams, case report writing, writing assignments, answering medical queries, and research assistance.

The AI bots known to the participants are depicted in Table 1.

**Table 1:** Awareness about the AI bots amongst the participants (n=377) *Multiple responses from participants.

Names of AI bots	Frequency *
ChatGPT (OpenAI, San Francisco, USA)	308
Google Gemini (Google LLC, Mountain View, USA)	306
Meta AI (Meta Platforms, Inc., Menlo Park, USA)	301
Grok (xAI, San Francisco, USA)	197
Microsoft Copilot (Microsoft Corporation, Redmond, USA)	180
Perplexity (Perplexity AI, San Francisco, USA)	101
DeepSeek (DeepSeek, Hangzhou, China)	92
Claude (Anthropic, San Francisco, USA)	33
Sora (OpenAI, San Francisco, USA)	02

**Figure 2:**
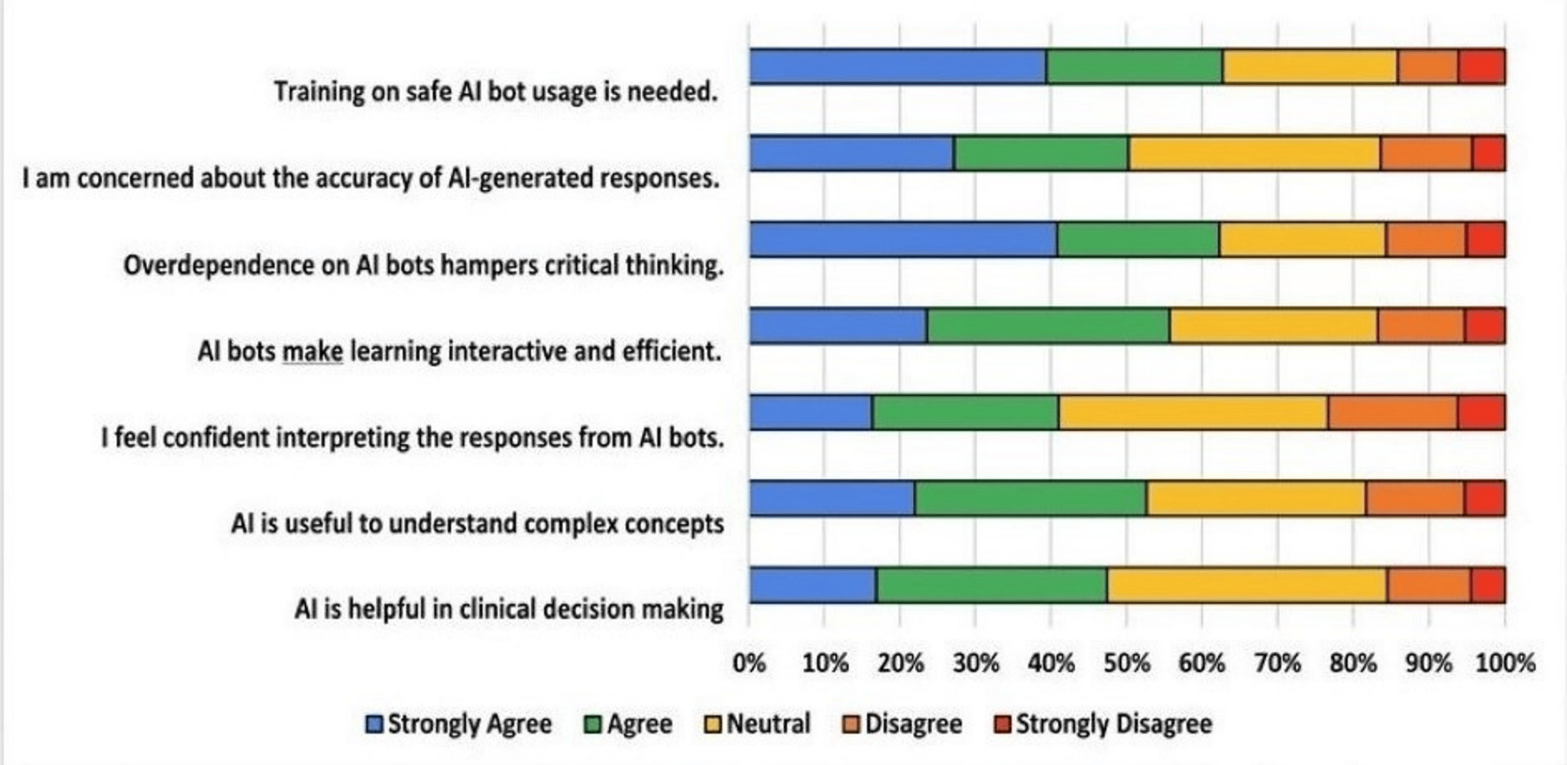
Bar chart showing the perception of participants regarding AI bots (n=377)

The participants showed their agreement regarding the need for training for AI bot usage (63%) and the concern regarding the accuracy of AI-generated responses (50.4%). Respondents also agreed that AI hampers critical thinking (62.3%) and regarding interpreting the responses from AI bots (41%). There was an agreement on understanding complex concepts (52.8%) and making clinical decisions using AI (48%) (Figure 2).

**Figure 3:**
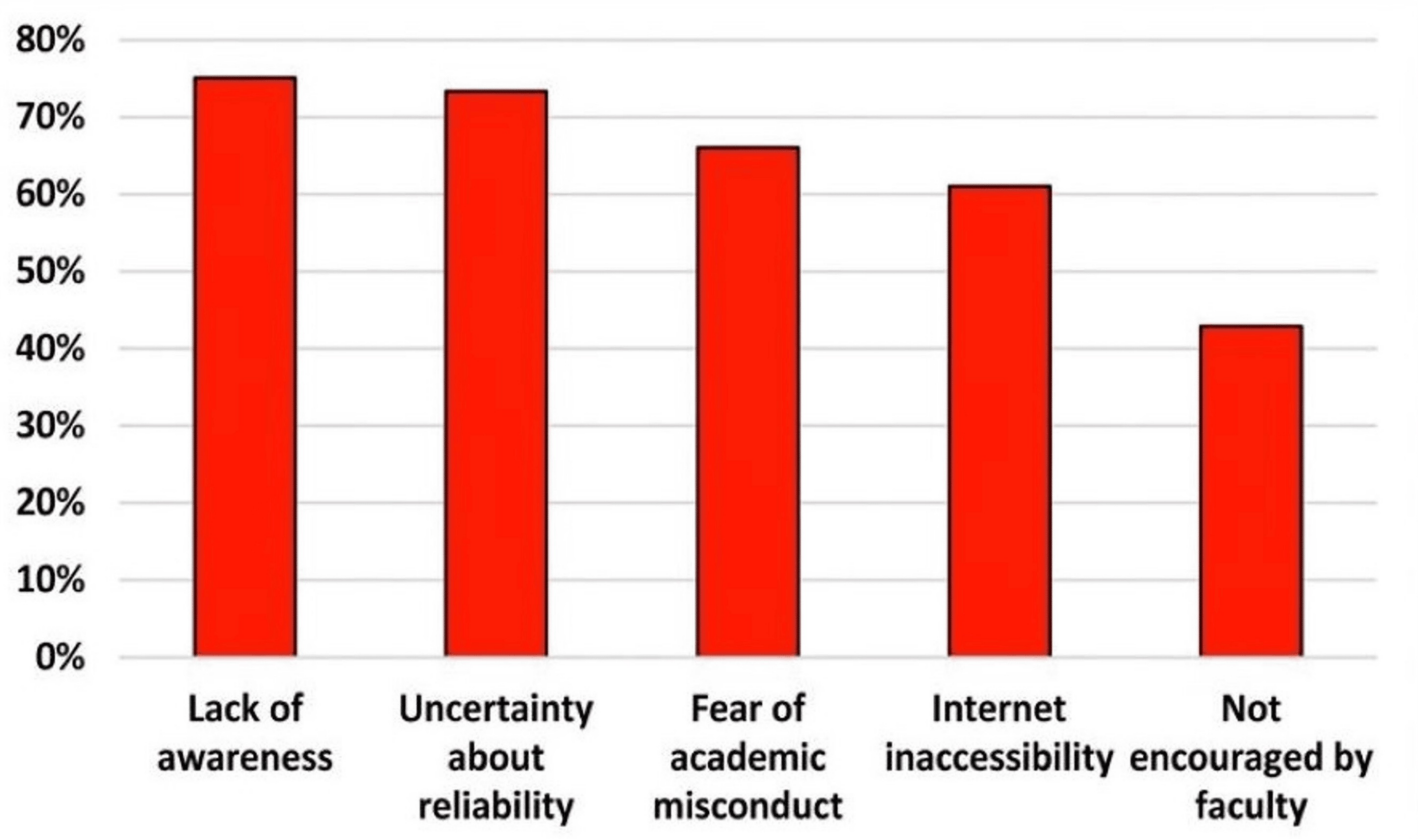
Bar chart showing the barriers in using AI bots among participants (n=377)

The barriers and facilitators in AI use among medical trainees and professionals were assessed, and it was found that major barriers were a lack of awareness about the AI bots available, reliability issues, fear of academic misconduct, internet connectivity, and non-endorsement by faculty members (Figure 3).

The factors recognized as facilitators by study participants are listed in Table 2.

**Table 2:** The list of facilitators for AI bot use as per the participants (377) *Multiple responses from participants

SN.	Facilitators	Frequency *
1.	Institutional guidelines	227
2.	Faculty capacity building programs	112
3.	Orientation workshops	97
4.	Demonstration sessions	90
5.	Peer learning & mentorship	85
6.	Ethical guidelines and code of conduct	66
7.	Integration in the curriculum	26

The responses from participants for enhancing medical education and clinical skills using AI bots suggested that the AI helps in getting a diagnosis in just a click, is easily approachable, saves time, is always available, can solve even trivial doubts, etc. But at the same time, participants also shared that the threats posed by AI bots in the current scenario are that life could become more dependent, can hamper the critical thinking of medical professionals, and unethical practices may flourish in the absence of stringent guidelines.

## Discussion

The cross-sectional study was conducted in a tertiary care center of southern Rajasthan, India, where nearly 377 medical trainees and professionals participated (response rate 37%). The participants were grouped as per their academic position, like consultants (10.6%) and students (89.4%). The current study is unique in its introduction and in capturing the perception of faculty regarding AI bot usage. In the previous studies, only students were selected as subjects, as compared to the current study [3,5-11]. The gender-wise distribution is in consensus with the previously conducted studies, where male and female participants were in a 1:1 ratio [3,5]. The awareness about AI was found to be 82% in the current study, which is comparable with the results in previously conducted studies by various authors [3,5,7,11]. The most commonly used AI bot was ChatGPT (OpenAI, San Francisco, USA) in the present study, and the same results were found in another study conducted in China in 2024 [6].

The participants in the current study stated that the orientation workshops or demonstrations should be held to train them for AI usage, and a similar response was seen among the participants from the previous study. The perception of participants regarding AI bots was also in consensus with previous authors, where it was found that AI aids in clinical diagnosis making, which was found in the current study too [12-14].

The findings indicate that although participants perceived AI as a useful tool in academic work, its use is limited by several important barriers. A major concern was the lack of awareness about appropriate and ethical AI usage, which reflects insufficient institutional policies or clear guidelines. This uncertainty often leads to hesitation among users despite their willingness to adopt the technology.

The findings suggest that while participants widely acknowledged the usefulness of AI in academic work, its adoption remains constrained by several key barriers. A prominent concern was the lack of awareness regarding appropriate and ethical use, which reflects the absence of clear institutional policies and guidelines. This ambiguity often creates hesitation among users, even when they are inclined to use such technologies.

Concerns about the reliability and authenticity of AI-generated content were also frequently expressed. Since AI responses are based on existing datasets, participants questioned their accuracy and contextual relevance, particularly in academic and clinical settings where evidence-based information is critical. In addition, fear of academic misconduct emerged as a significant issue, with many participants uncertain whether AI use might be perceived as plagiarism or unethical assistance. Practical challenges, including limited internet access and insufficient encouragement from faculty, further limited its effective utilization.

At the same time, participants identified several facilitators that could enhance AI adoption. The need for well-defined institutional guidelines was strongly emphasized to ensure clarity and promote ethical practices. Faculty support was also considered crucial, as endorsement from educators can build confidence and normalize AI use in learning environments. Furthermore, orientation workshops and demonstration sessions were suggested as effective strategies to improve awareness and provide hands-on experience, enabling users to better understand both the potential and limitations of AI tools.

Participants also reflected on the broader role of AI in medical education and clinical practice. Many appreciated its ability to provide quick, accessible, and time-saving solutions, including rapid diagnostic insights and clarification of even basic doubts. However, concerns were raised about overdependence on AI, which could negatively impact critical thinking and clinical reasoning skills. There was also apprehension that, without strict regulations, unethical practices might increase. Overall, while AI is seen as a valuable support tool, its effective integration requires a balanced approach that emphasizes ethical use, critical thinking, and supportive institutional frameworks.

Limitations

The study’s findings may be limited by its cross-sectional design and reliance on self-reported data, which can introduce bias and restrict causal interpretation. Additionally, the results are limited to medical professionals and trainees and may not be generalizable beyond this specific group or to the wider population.

## Conclusions

In conclusion, the present study highlights that while AI is increasingly recognized as a valuable tool in medical education and clinical practice, its adoption is influenced by a combination of enabling factors and perceived challenges. The findings demonstrate a high level of awareness and acceptance among participants, with AI being appreciated for its accessibility, time-saving nature, and support in clinical decision-making. The inclusion of both students and faculty adds strength to the study by providing a broader perspective on AI usage in academic settings.

However, the study also underscores important barriers, including a lack of clear institutional guidelines, concerns regarding the reliability and authenticity of AI-generated content, and fear of academic misconduct. These challenges contribute to hesitation in adoption despite the acknowledged benefits. Additionally, issues such as limited infrastructure and insufficient faculty encouragement further restrict optimal utilization.

The identification of facilitators such as institutional policies, faculty endorsement, and structured training initiatives highlights the need for a supportive ecosystem. Addressing these gaps through clear guidelines, capacity building, and ethical frameworks is essential. Overall, a balanced and regulated approach is required to integrate AI effectively, ensuring that it enhances learning and clinical skills without compromising critical thinking or professional integrity.

## Appendices

Appendix 1

Questionnaire

 A Study to Assess the Awareness and Attitudes towards Artificial Intelligence (AI) tools among Medical Trainees and Practitioners

Demographic Information

1. Age: _____ years

2. Gender: ☐ Male ☐ Female ☐ Other ☐ Prefer not to say

3. Current Academic/Professional Status: ☐ MBBS student ☐ Postgraduate (MD/MS/DNB) ☐ Consultant/Faculty

4. Year of study (if student): ☐ 1st ☐ 2nd ☐ 3rd ☐ Final MBBS ☐ Internship (Leave blank if not applicable)

5. Specialty (for postgraduates/consultants): _______________

6. Have you received any formal or informal training on Artificial Intelligence (AI)? ☐ Yes ☐ No

Awareness and Use of AI Bots

7. Have you heard of AI bots used in medicine or education? ☐ Yes ☐ No

8. Which AI bots are you aware of? (Select all that apply) ☐ ChatGPT ☐ Google Gemini ☐ Meta AI ☐ Microsoft Copilot ☐ Other: __________ ☐ None

9. Have you ever used an AI bot for academic or clinical purposes? ☐ Yes ☐ No

10. If yes, how frequently do you use it? ☐ Daily ☐ Weekly ☐ Occasionally ☐ Rarely

11. For what purposes have you used an AI bot? (Select all that apply):

- ☐ Studying medical concepts

- ☐ Answering clinical queries

- ☐ Preparing for exams

- ☐ Writing assignments/research

- ☐ Creating presentations or case reports

- ☐ Simulating patient interaction or decision-making

-☐ Research purposes

- ☐ Other: ___________

Attitudes Towards AI Bots

Please indicate your level of agreement (1 = Strongly Disagree to 5 = Strongly Agree):

12. AI bots are useful for understanding complex medical concepts.

13. I find AI bots helpful in clinical decision-making or preparation.

14. I feel confident interpreting the responses from AI bots.

15. AI bots can make learning more interactive and efficient.

16. Overdependence on AI bots may hamper critical thinking.

17. I am concerned about the accuracy of AI-generated medical content.

18. There is a need for training or guidance on safe AI bot usage.

Barriers and Facilitators

19. What prevents you from using AI bots more often? (Select all that apply):

- ☐ Lack of awareness

- ☐ Uncertainty about reliability

- ☐ Fear of academic misconduct

- ☐ No internet/device access

- ☐ Not encouraged by faculty

- ☐ Other: ___________

20. What would encourage you to use AI bots more effectively?

- ☐ Workshops or orientation

- ☐ Faculty endorsement

- ☐ Demonstrations of medical applications

- ☐ Institutional guidelines

- ☐ Other: ___________

Open-Ended Questions

21. How do you think AI bots can enhance medical education or practice in your setting?

22. Do you have any concerns or suggestions about integrating AI tools in medical training or decision-making?
